# Soil Nitrogen Content Detection Based on Near-Infrared Spectroscopy †

**DOI:** 10.3390/s22208013

**Published:** 2022-10-20

**Authors:** Baohua Tan, Wenhao You, Shihao Tian, Tengfei Xiao, Mengchen Wang, Beitian Zheng, Lina Luo

**Affiliations:** 1 School of Science (School of Chip Industry), Hubei University of Technology, Wuhan 430068, China; 2 National “111 Research Center” Microelectronics and Integrated Circuits, Hubei University of Technology, Wuhan 430068, China; 3 School of Physical Education, Hubei University of Technology, Wuhan 430068, China

**Keywords:** near-infrared spectroscopy, soil nitrogen content, random forest algorithm

## Abstract

Traditional soil nitrogen detection methods have the characteristics of being time-consuming and having an environmental pollution effect. We urgently need a rapid, easy-to-operate, and non-polluting soil nitrogen detection technology. In order to quickly measure the nitrogen content in soil, a new method for detecting the nitrogen content in soil is presented by using a near-infrared spectrum technique and random forest regression (RF). Firstly, the experiment took the soil by the Xunsi River in the area of Hubei University of Technology as the research object, and a total of 143 soil samples were collected. Secondly, NIR spectral data from 143 soil samples were acquired, and chemical and physical methods were used to determine the content of nitrogen in the soil. Thirdly, the raw spectral data of soil samples were denoised by preprocessing. Finally, a forecast model for the soil nitrogen content was developed by using the measured values of components and modeling algorithms. The model was optimized by adjusting the changes in the model parameters and Gini coefficient (∆Gini), and the model was compared with the back propagation (BP) and support vector machine (SVM) models. The results show that: the RF model modeling set prediction R^2^_C_ is 0.921, the RMSEC is 0.115, the test set R^2^_P_ is 0.83, and the RMSEP is 0.141; the detection of the soil nitrogen content can be realized by using a near-infrared spectrum technique and random forest algorithm, and its prediction accuracy is better than that of the BP and SVM models; using ∆ Gini to optimize the RF modeling data, the spectral information of the soil nitrogen content can be extracted, and the data redundancy can be reduced effectively.

## 1. Introduction

With the continuous development of technology, the national population base and the demand for quality food are increasing. Agricultural production is the foundation of ensuring national living standards and protecting food security. Therefore, improving agricultural production efficiency is the current top priority.

Applying agricultural chemical fertilizers to crops is one of the most important methods used to improve agricultural production at present. Pesticides and fertilizers can increase the nutrients in the soil, thereby boosting the production of crops. However, imprecise fertilization can have negative effects. Too little fertilizer application will result in an insufficient nutrient supply for crops, which cannot meet the growth needs, consequentially resulting in low yields and affecting agricultural production efficiency. Excessive fertilization will cause excess nutrients to deposit in the soil. These nutrients not only disrupt the physical properties and nutrient balance of the soil, but also lead to an excess of metals and an infestation of harmful bacteria. In the long run, soil productivity will decline, and it will lead to eutrophication of groundwater, rivers and lakes, causing serious pollution to the environment. Therefore, it is very important to obtain soil composition information quickly and accurately.

In the development stage of precision agriculture in the 21st century, ensuring soil safety protects the foundation of crop production. The detection of soil components can not only effectively control the nutrient requirements of crops, but can also avoid the abuse of chemical fertilizers that cause environmental pollution. The nitrogen content in soil is a very important index of soil nutrition. The content of nitrogen in soil is the main composition of plant proteins, nucleus acid, enzymes, and so on. It is also the foundation of life activity and genetic variation. Without nitrogen, the growth of crops is slow and there are few branches and leaves. Too high a nitrogen content often manifests as breakage and a low yield of crops [1].

The traditional methods for detecting the nitrogen content in soil are: the Kjeldahl method [2,3], ion electrode [4], and ultraviolet spectrophotometry. Although the test results are accurate, they have the disadvantages of a long test process, being time-consuming, pollution, inconvenient operation and promotion, and high requirements regarding the quality of operators. Due to unstable factors such as geography and space, remote sensing methods [5,6,7] are not feasible to use to obtain the soil nitrogen content. At present, the means of obtaining agricultural soil information is relatively simple. The information technology gap for the rapid detection of the soil component content is the bottleneck of modern precision agriculture [8]. There is an urgent need for a rapid, easy-to-operate, non-polluting, and convenient soil composition detection method.

Infrared spectroscopy has become a mature major chemical analysis technology [9]. At present, it has been rapidly developed and used in important industries, such as food [10,11,12], chemical industry [13,14,15], and medicine [16,17,18,19]. Near-infrared spectroscopy technology has the following characteristics: a fast speed, convenient detection, low cost, no pollution in the detection process, wide detection range, high detection efficiency, non-destructive, able to simultaneously determine multiple groups, etc. With the rapid development of computer application technology, chemometrics methods, statistical theory, and the high integration of multi-disciplinary technologies have been put forward, and their feasibility for NIR detection has been verified [20,21,22,23,24]. At the same time, random forest (RF) is a common machine learning method that is usually used to deal with classification [25,26,27] and regression [28,29,30] problems. This method improves the prediction accuracy without significantly increasing the computational complexity. In addition, the results are more robust to missing data and unbalanced data. This paper uses near-infrared spectroscopy and RF to realize a low-cost, green, and rapid soil composition detection system.

## 2. The Related Work

Fang et al. [31] (2015) collected the spectral data of 394 farmland soil samples and used a least squares support vector machine (LS-SVM) model to prove that the detection of soil components can be achieved by using near-infrared spectroscopy. Li et al. [32] (2017) used visible–near-infrared spectroscopy to predict nitrogen, phosphorus, and potassium concentrations in non-isotropic soils, which could reduce the cost of the rapid determination of soil nutrients. Chen et al. [21] (2018) concluded that it was possible to optimize and integrate the FT-NIR analysis model with suitable stoichiometric methods. Compared with the traditional model, the BPN-DL model showed its superiority in the training and testing of soil nutrient component models. Xiang et al. [33] (2019) used the preprocessing algorithm to denoise the original spectral data of the near-infrared spectrum collected from the soil and showed that Savitzky–Golay convolution smoothing and the least squares support vector machine use the spectral data in the 400–850 nm band. The established soil phosphorus content prediction model has the best effect, and it also proves that the NIR spectroscopy technology combined with the LS-SVM regression algorithm used to establish the soil phosphorus content prediction model can realize the detection of the soil phosphorus content. Wang et al. [34] (2021) demonstrated that VIS/NIRS has a large potential to detect black soil characteristics in real time. Qiao et al. [22] (2022) demonstrated that SVD-CNN has a good prediction and generalization ability in soil component content detection.

Xu et al. [35] (2017) showed that the convolution smoothing competitive adaptive-random forest model established by near-infrared spectroscopy has a high prediction accuracy for the sugar content and acidity of red grapes. Li et al. [27] (2018) used the near-infrared spectral detection technology to study the research on the non-destructive detection of fruit sugar, and showed the feasibility of the fruit near-infrared non-destructive detection model established by the random forest algorithm. Li et al. [28] (2019) proved that NIR spectroscopy combined with RF is an effective means to rapidly detect the methanol content in methanol gasoline. Kartakoullis et al. [29] (2019) showed that fat and moisture content can be detected by building a random forest model using a full spectrum over a wide temperature range using a smartphone-based spectrometer with a good detection accuracy (RPD > 7), comparable to the accuracy of benchtop spectrometers. The results of Liu et al. [36] (2020) demonstrated that NIR spectroscopy combined with the random forest algorithm is a quick and non-destructive method used to detect sunset yellow in cream. Du et al. [37] (2021) demonstrated that the ADA content in flour can be precisely determined by NIR combined with the random forest algorithm.

Liu et al. [30] (2017) showed that the RF algorithm is able to strongly optimize the information of soil organic matter, reduce the dimension of spectral data, and optimize the model. At the same time, the experimental study also showed that the near-infrared spectroscopy technology combined with the RF algorithm can realize the detection of the soil organic matter content. Shahrayini et al. [38] (2020) showed that VIS-NIR has the ability to detect electrical conductivity (ECe), organic carbon (OC), and texture (including sand, silt, and clay) classifications. Cui et al. [39] (2021) adopted various preprocessing techniques and band screening algorithms, and then combined the least squares method and random forest to establish an organic matter prediction model used to measure the true value of soil organic matter. The results indicate that the competitive adaptive reweighting (CARS) random forest (RF) model is the best. The author designed a portable soil organic matter content detector based on CARS-RF. Luo et al. [40] (2021) showed that near-infrared optical disc technology is effective and fast in the prediction of the content of organic matter in soil. Hong et al. [41] (2021) showed that Cd-contaminated soil leads to a decrease in spectral reflectance. Combined with the CR preprocessing-SMOTE strategy-RF algorithm, the prediction model is the best, and the verification accuracy is the highest (kappa = 0.74). This model can realize the detection of soil Cd. This research provides a theoretical basis for rapidly identifying and monitoring soil cadmium pollution in urban and suburban areas.

To sum up, the NIR spectroscopy detection technology has been widely used in the rapid detection of soil components, but the prediction accuracy of the obtained model still has room for improvement and the model can also be optimized. The research field of near-infrared detection based on a random forest is very wide, and the prediction accuracy is high. In terms of soil detection, random forests are often used in soil regression and classification studies, such as organic matter and metal element content prediction and soil texture classification. The accuracy of the results can be improved, the model still has room for optimization, and many other components can also be predicted. Therefore, this paper combines infrared spectroscopy technology and the random forest algorithm to study soil composition prediction.

## 3. Materials and Methods

### 3.1. Materials

The material used in the experiment was the soil of the Xunsi River basin near Hubei University of Technology. Soil samples were collected from 143 sampling points. The topsoil was removed with a small shovel and soil was taken at 10–20 cm. The mass of the sub sample taken was 200 g. After the soil sample was retrieved, the fresh wet soil sample was spread on a clean storage box or paper and broken into pieces. Then, it was spread into a thin layer of approximately 2 cm and placed in a ventilated place indoors in the light to dry. Impurities such as stones, roots, leaves, and insects were removed. The soil samples after drying were packed in beakers and placed in an electric blast dryer for dehydration. The dehydrated soil samples were then ground and passed through an 80-mesh sieve because the soil particle size will affect the detection accuracy. After weighing with an electronic balance, it was put in a clean plastic bag and labelled. The weight distribution of the collected and subpackaged soil samples was 8.205–9.385 g, with an average value of 8.646 g and a mean square error of 0.246.

### 3.2. Measurement of Actual Nitrogen Content

The experimental principle of the Kjeldahl method is as follows:

A catalyst and concentrated sulfuric acid are added to the soil sample and stirred well. Organic nitrogen in the soil will be converted to inorganic ammonium salts. The ammonium salt is then converted to ammonia under alkaline conditions, and the ammonia in solution is absorbed by boric acid. Finally, the prepared standard concentrated sulfuric acid and indicator are used. The solution is titrated and the standard amount of concentrated sulfuric acid at the time of titration is recorded. The nitrogen content of the soil is calculated by the formula.

In this paper, soil nitrogen content was determined by Kjeldahl method.

Soil digestion: firstly, approximately 1.0 g of the soil sample to be tested was weighed and put into the bottom of the dried digestion tube for soil digestion. Secondly, 5 mL concentrated sulfuric acid, 1 mL distilled water, and 2 g catalysts were added to the tube. Then, it was mixed and shaken well. Thirdly, the bottom of the cooking tube was put on the cooking stove and heated on low heat. The temperature was controlled to keep the soil liquid in the digestion tube slightly boiling. The heating temperature and time should not be too high to prevent the loss of nitrogen content in the soil. Fourthly, during the digestion process, the sulfuric acid vapor was condensed and refluxed at the third position of the nozzle. Fifthly, we waited until the color of the soil liquid changed to gray-white and slightly green, and it was cooked for another hour. Finally, blank experiments were carried out in the same manner.

Distillation: distillation uses an automatic Kjeldahl nitrogen analyzer. Firstly, the distillation pipeline was cleaned and the instrument parameters were set. Secondly, the previously configured reagents were added separately to each set point. Thirdly, the Kjeldahl nitrogen analyzer was preheated until the instrument detection was stable. Finally, the liquid to be tested was distilled.

Titration: a mixed indicator was added to the receiving solution and titrated with 0.02 mol/L sulfuric acid standard solution. The blank value of Kjeldahl nitrogen determination cannot exceed 0.8 mL. If the blank value is too high, it means that there is a systematic error in the instrument and the sample is not digested well. It was titrate to the end point color; the end point color is gray-red, and the excess titration color is wine red.

After the above three steps, the actual value of nitrogen content has been measured.

Equation (1) shows the results of the calculation of the nitrogen content in the soil.

(1) $$N(g/kg)=\frac{(V_{1}-V_{0})\times C\times 14.01\times 1000}{m}\times 0.001$$

where V_1_ is the volume of sulfuric acid standard titrant consumed in mL of the test solution. C is the sulfuric acid standard titration solution in mol per liter (mol/L). V_0_ is the volume of the standard titrant used in ml. m is the weight of the dry soil sample (g). In addition, 14.01 is the molar weight of the nitrogen atom (g/mol)

Materials required in the experiment included:

1. Freshly prepared deionized water;
2. Premium pure concentrated sulfuric acid: ρ(H_2_SO_4_) = 1.84 g/mL;
3. Mixing catalysts: selenium powder, copper sulfate pentahydrate (CuSO_4_•5H_2_O), and potassium sulfate (K_2_SO_4_) were mixed in a ratio of 100:10:1;
4. Premium pure sodium hydroxide (NaOH);
5. Premium pure boric acid (H_3_BO_3_);
6. Sodium hydroxide solution: ρ(NaOH) = 400 g/L;
7. Methyl red-bromocresol green mixed indicator: 0.1 g of methyl red was added to 100 mL of ethanol solution, then 0.5 g of bromocresol green was weighed into the mixture and mixed well;
8. A total of 20 g/L boric acid-indicator solution: 2 g of boric acid was added to 100 mL of distilled water, and then 3 to 4 drops of methyl red-bromocresol green mixed indicator were added to the mixture. The mixed solution was adjusted to Ph = 4.8, and the color changed to slightly purple-red;
9. Standard stock solution of sulfuric acid: c(H_2_SO_4_) = 0.02 mol/L.

The actual nitrogen content distribution of 143 soil samples collected was 0.609–2.104 g/kg, with an average value of 1.471 g/kg. Among 143 samples, 43 samples were randomly selected as the test set, and the remaining 100 samples were the modeling set. The ratio is 3:7.

### 3.3. Spectral Acquisition

**Figure 1:**
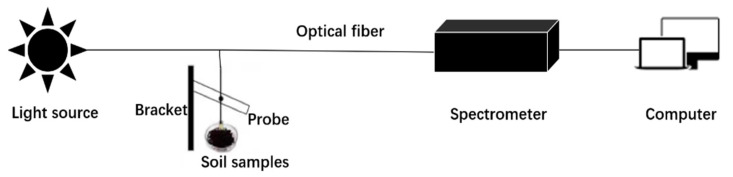
Schematic diagram of the experimental system.

The NIR Quest (256–2.5) NIR spectrometer of Ocean Optics, optical fiber measurement equipment, HL-2000 tungsten-halogen light source, and computer were used to build a near-infrared spectrum system. The system is shown in Figure 1.

The detection principle of near-infrared spectroscopy is as follows.

When a molecule is irradiated by infrared light, resonance occurs only when the vibrational frequency of the group in the molecule is the same as the vibrational frequency of the radiation photon. After resonance, the dipole moment of the molecule changes, and the group absorbs infrared radiation photons and transitions. The near-infrared absorption spectrum in the table is the absorption region of some components, and there are also absorption regions of some metal-inorganic/organic bonds (such as potassium, cadmium, phosphorus). However, these absorptions are caused by the different components of the measured substances. The shift in the infrared absorption band is also the reason why most substances cannot determine the absorption spectral band. The absorption of material components on the near-infrared spectrum provides a theoretical basis for the qualitative and quantitative detection of nitrogen, potassium, organic matter, and other substances in soil components using near-infrared spectroscopy.

**Figure 2:**
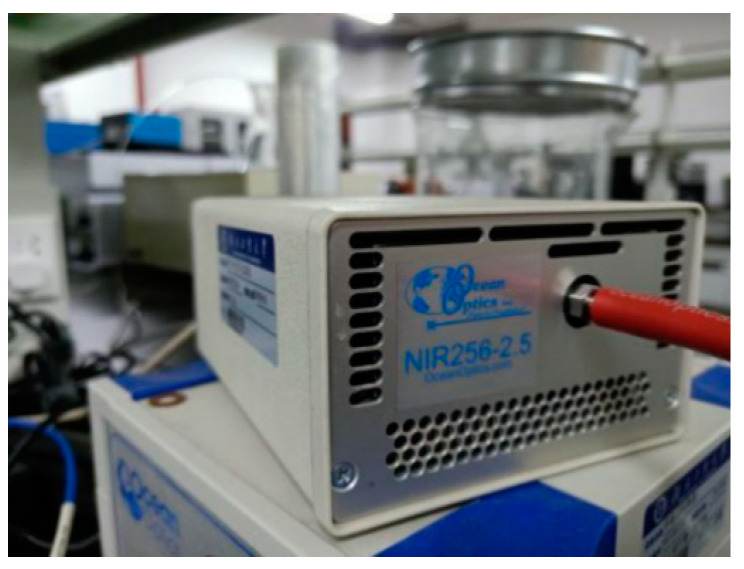
Near-infrared spectrometer.

The spectrometer used in this study was the NIR Quest (256–2.5) NIR spectrometer of Ocean Optics, the appearance of which is shown in Figure 2. The light source was HL-2000 halogen tungsten light source. The parameters are shown in Table 1.

The principles of system construction are as follows. A tungsten halogen lamp is irradiated on the soil sample through a fiber optic probe. The near-infrared light interacts with the interior material of the soil sample, and the rest of the near-infrared light, which carries information about the composition of the soil, is collected by the spectrometer. Spectra collected by the spectrometer are presented by computer-enabled instrument software, and the spectrum data are stored in the computer. Table 2 is the experimental parameter setting table.

**Figure 3:**
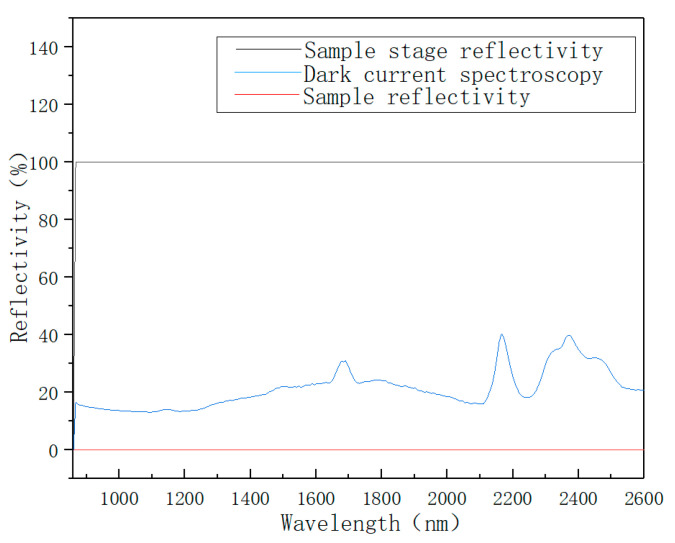
Instrument debugging before sample collection.

When collecting spectra with Spectrasuite, the light source was blocked and the background noise was tested. “Dark Bulb” was clicked to record background noise. Then, “Subtract Dark Bulb” was clicked to deduct background noise during the test. Then, the light source was turned on and “Bright Bulb” was clicked to collect the raw spectrum. The reflectivity should be 100% when the probe is aimed at an empty petri dish, as shown in Figure 3. In this way, the reflection spectra collected by the spectrometer are all of the remaining spectra after the near-infrared light reacts with the soil sample.

**Figure 4:**
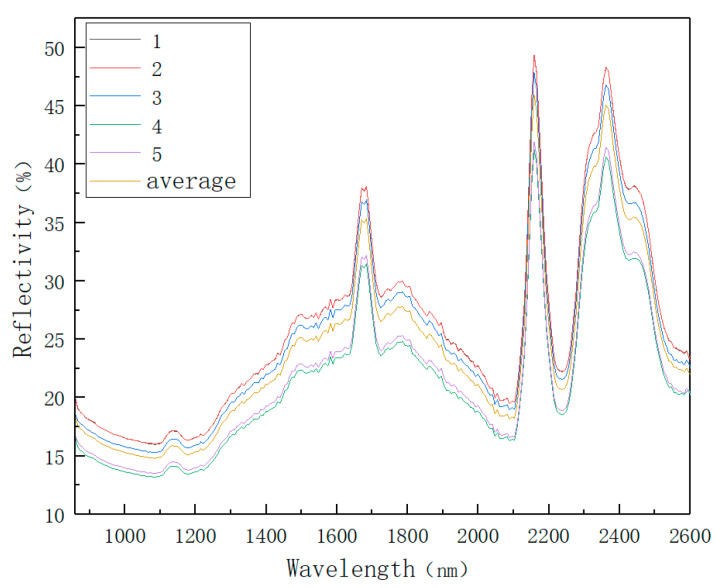
Five-point sampling spectrum of the sample.

In order to reduce the influence of operation error and instrument error on the results, five points near the center of each soil sample were scanned ten times each. The result was the average of ten numbers. The spectral data representing this point are shown in Figure 4.

**Figure 5:**
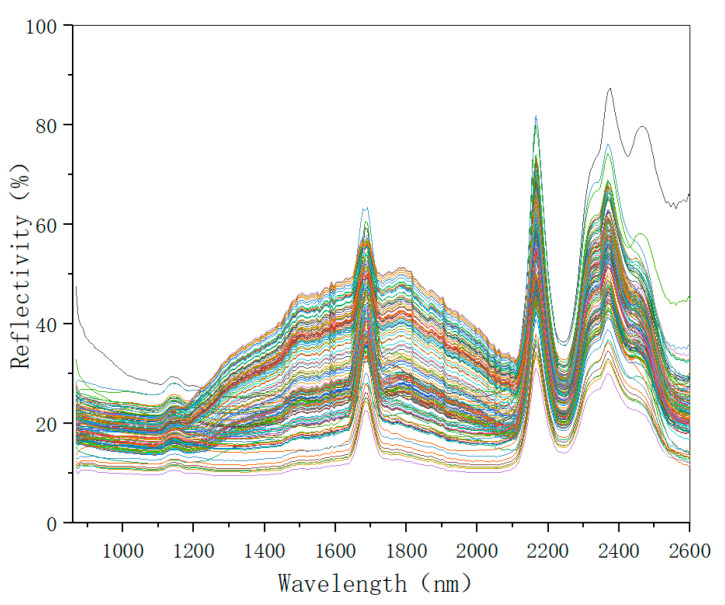
Original spectrogram of soil sample.

The mean of the spectral data of the five points represents the soil sample spectrum data. Normalizing the procedure as described above and changing the background spectrum every hour. Some obviously problematic data points were eliminated by preset conditions. The spectral data of 143 soil samples were collected by setting the parameters of the experimental instrument and controlling the collection environment. The spectral data are shown in Figure 5.

From the original spectra of the soil sample, it can be seen that the overall spectral trend of each sample is consistent at 800–2600 nm. However, there are some differences in the spectra of different samples. Because of the difference in the content of each soil sample, the spectrum information also contains many sample components, and it is feasible to build a NIR detection model.

### 3.4. Spectral Denoising

**Figure 6:**
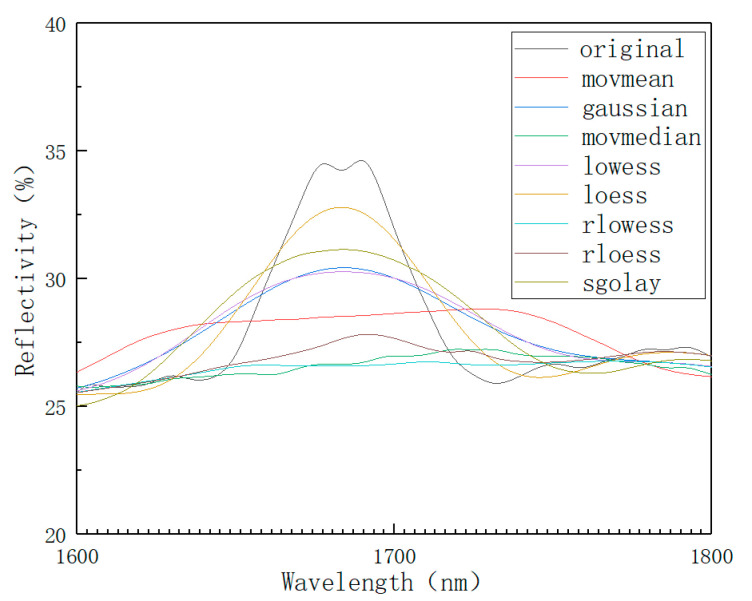
A 1600 nm~1800 nm band smoothing comparison chart.

In the process of spectral data collection, optical noise will be generated due to operation errors, instrument errors, environmental errors, etc. Therefore, after the data spectrum is collected, it is necessary to perform data smoothing and noise reduction to reduce the interference of light noise and improve the modeling accuracy. Common smooth noise reduction methods are as follows [42]: moving average (movmean), Gaussian filter (Gaussian), moving median (movmmedian), local weighted regression (lowess), local polynomial regression fitting (loess), robust local weighted regression (rlowess), robust local polynomial regression (rloess), and least squares smoothing filter (sgolay). A variety of spectral denoising methods were used to denoise the collected original spectra, and the spectral data in the 1600–1800 nm band were observed. The result is shown in Figure 6.

As can be seen from Figure 6, the data peaks after moving average and robust local weighted regression processing are significantly lower than the original data and are also lower than other smoothing methods. If robust local weighted regression and moving average methods are used, spectral data will be distorted. The error of the result is large, so it is excluded. However, other methods cannot judge whether the effect is good or bad from the figure, so the smoothing effect was comprehensively evaluated by introducing the root mean square error (RMSE) evaluation index. The smaller the calculated RMSE value, the better the selected smoothing method.

**Figure 7:**
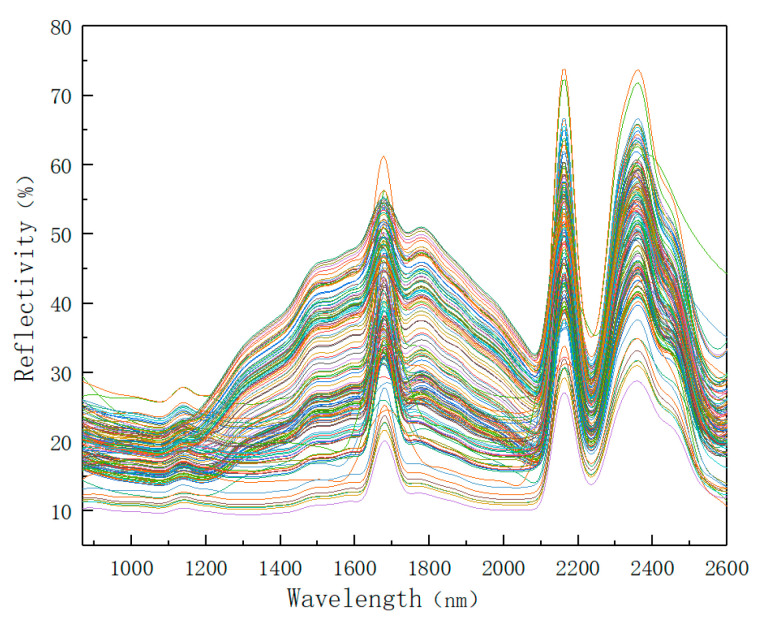
A total of 143 spectral graphs smoothed by the loess method.

As shown in Table 3, the root mean square error (RMSE) value of local polynomial regression fitting is the smallest. This means that the smoothing effect of the local polynomial regression fitting (loess) method is the best. Therefore, the algorithm used in this paper was loess smoothing denoising. Figure 7 below shows the spectral data of 143 soil samples after smoothing and denoising. It can be seen from the figure that the denoised spectral image appears as smoother.

### 3.5. RF Regression Modeling Methods

**Figure 8:**
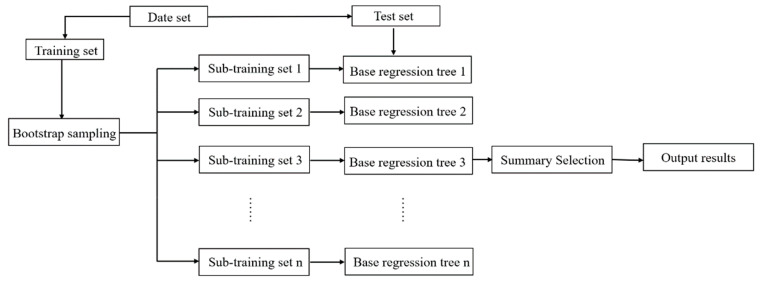
Schematic diagram of the random forest algorithm model.

Random forest is a (parallel) ensemble algorithm composed of decision trees. Random forest completes classification and regression by integrating classification and regression trees (CART). Its modeling is shown in Figure 8. The principle of random forest is as follows: applying resampling method can continuously generate new training set data. N decision trees are generated by randomly sampling N samples from all training set data. The splitting feature of the decision tree is generated by random extraction. All CART regression trees are trained up to the maximal depth of the tree, and the random forest model is formed.

Regression analysis based on the stochastic forest algorithm was used to obtain the corresponding regression value by dividing all of the nodes in the forest. Then, the average estimation of the regression value for all decision trees was completed. This mean represents the predicted value of the random forest model. The schematic diagram of the random forest algorithm modeling is shown in Figure 8.

### 3.6. Model Optimization

#### 3.6.1. Parameter Optimization

RF regression model contains a large number of decision trees. Each decision tree will decompose the variable into different leaf nodes to achieve its natural growth. Ntree and NSV are the most important factors in RF model. RF model can be optimized by optimizing the number of decision trees (Ntree) and the number of nodes (NSV).

#### 3.6.2. Wavelength-Based Filtering Random Forest Model Optimization

In the near-infrared spectral data modeling, the random forest model predicts the composition of each soil sample based on the soil spectral data. Different spectral data have different contributions to the split growth of the CART regression tree in the random forest modeling process. Different spectral features have different correlations with component content. Therefore, by comparing the relative contribution of each wavelength to the construction of the CART regression tree, the spectral data with high relative importance can be selected, thereby reducing data redundancy and model complexity and optimizing model prediction speed.

The Gini coefficient represents the contribution of each eigenvalue pair to the split growth process of the CART regression tree in random forest modeling. The smaller the Gini value at each node, the smaller the probability of feature error, and the higher the information purity. The greater the change in Gini above and below each node, the greater the contribution value and the greater the importance of the feature. When the RF model is established in the near-infrared spectrum, the Gini value of the reflection spectral feature of each wavelength is calculated. The calculation formula is shown in Equation (2).

(2) $$G(i)=1-\overset{N}{\underset{j=1}{\sum }}\left(\frac{a(i,j)}{\sum_{j=1}^{N}(a(i,j))}\right)$$

where a(i,j) represents the reflectance of the jth near-infrared spectral sample at the ith characteristic wavelength. For the spectral data measured for each soil sample, the Gini coefficient values of all its characteristic wavelengths were calculated. Then, the minimum value of the G value of the spectral sample point and the corresponding characteristic wavelength v can be obtained.

(3) [G(t),v]=min{G(i)∣i∈t}

Another Gini variation was introduced: ∆Gini. ∆Gini refers to the change in Gini during the splitting process of each node of the decision tree. For example, if a node is split into multiple nodes, the Gini value of the first node will also be divided into Gn (n = 1,2,3 … n). Then, ∆Gini is G − (G1 + G2 + … + Gn). The contribution importance of each feature wavelength in constructing the random forest CART regression tree was measured by the change in G [43]. The Gini coefficient values of all CART regression tree nodes before and after splitting and the mean ΔGini of each feature spectrum were jointly calculated. All characteristic wavelengths were optimized by ∆Gini to optimize the data optimization and the prediction accuracy of the RF regression model.

### 3.7. Other Modeling Algorithms

#### 3.7.1. Principle of Support Vector Machine (SVM)

In the process of support vector regression modeling, the regular regression function is f(x)=ω⋅x+b and the fitting accuracy ε was set. To better control the error, the relaxation factor $\xi_{i},\xi_{i}^{*}\left(\xi_{i},\xi_{i}^{*}\geq 0\right)$ was increased. It can become:

(4) $$\begin{array}{l} y_{i}-\omega \cdot x_{i}-b\leq \varepsilon +\xi_{i}\\ \omega \cdot x_{i}+b-y_{i}\leq \varepsilon +\xi_{{}_{i}}^{*} \end{array}$$

In Equation (4), b represents the deviation, and ω represents the normal vector of the hyperplane.

The regression optimization problem of SVM is to minimize $\frac{1}{2}\|\omega {\|}^{2}+C\cdot \sum_{i=1}^{I}\left(\xi_{i}+\xi_{i}^{*}\right)$, where C is the penalty coefficient. While in condition $\sum_{i=1}^{I}\left(a_{i}-a_{i}^{*}\right),0\leq a_{i},a_{i}^{*}\leq C,\;i=1,2\cdots I$, the calculation process becomes a dual problem. Its maximum objective function is expressed as Equation (5).

(5) $$W\left(a,a^{*}\right)=-\varepsilon \overset{I}{\underset{i=1}{\sum }}\left(a_{i}+a_{i}^{*}\right)+\overset{I}{\underset{i=1}{\sum }}\left(a_{i}^{*}-a_{i}\right)\cdot \left(a_{j}^{*}-a_{j}\right)\left(x_{i}\cdot x_{j}\right)$$

In the formula, ai is not all 0, and the support vector is the corresponding sample data. Then, the regression function is:

(6) $$f(x)=w\cdot x+b=\frac{1}{2}\overset{I}{\underset{i,j=1}{\sum }}\left(a_{i}^{*}-a_{i}\right)\left(x_{i}\cdot x_{j}\right)+b^{*}$$

In Equation (6), a_i_ represents the optimal solution of the dual problem and b* represents the optimal deviation of the dual problem.

In nonlinear problems, the low-dimensional nonlinear problem is only converted into a high-dimensional linear problem. The low-dimensional kernel function K(x_i_,x_j_) is used to replace the high-dimensional inner product operation. Therefore, the objective function becomes as shown in Equation (7).

(7) $$W\left(a,a^{*}\right)=-\varepsilon \overset{I}{\underset{i=1}{\sum }}\left(a_{i}+a_{i}^{*}\right)+\overset{I}{\underset{i=1}{\sum }}\left(a_{i}-a_{i}^{*}\right)-\frac{1}{2}\overset{I}{\underset{i,j=1}{\sum }}\left(a_{i}^{*}-a_{i}\right)\cdot \left(a_{j}^{*}-a_{j}\right)k\left(x_{i}\cdot x_{j}\right)$$

The corresponding regression function is transformed into Equation (8).

(8) $$f(x)=\omega \cdot x+b=\overset{I}{\sum }\left(a_{i}^{*}-a_{i}\right)k\left(x_{i},x_{j}\right)+b$$

Because SVM has a good effect on linear and nonlinear data regression.

#### 3.7.2. Principle of Back Propagation Neural Network

**Figure 9:**
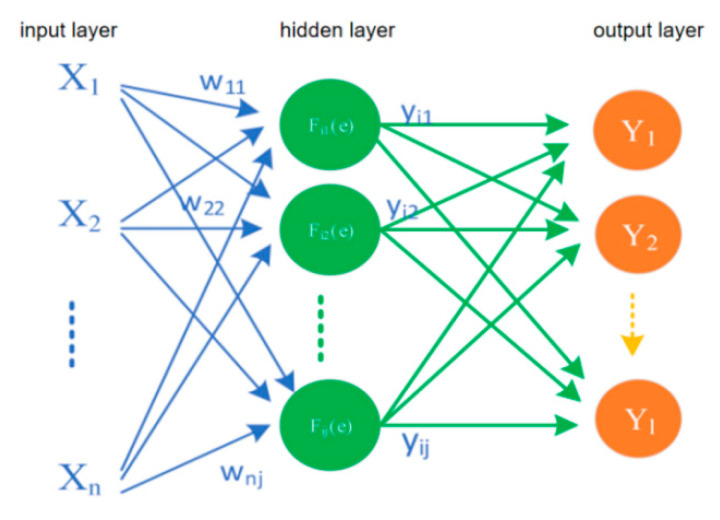
Schematic diagram of back propagation neural network.

Back propagation (BP) neural network is the most classic and successful algorithm in neural network. The BP network structure is mainly composed of input layer, hidden layer, and input layer. The schematic diagram of its model construction is shown in Figure 9.

**Figure 10:**
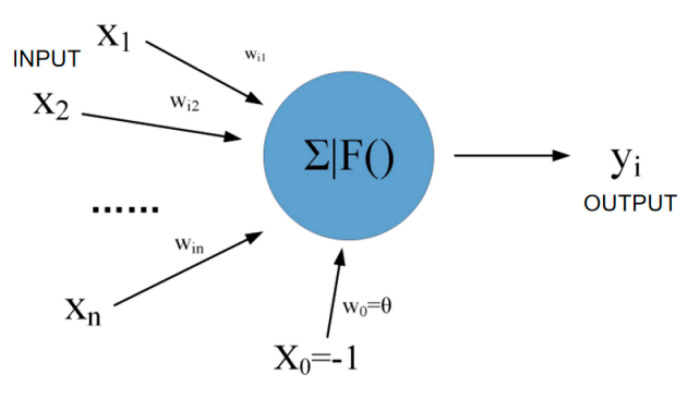
Schematic diagram of back propagation neuron calculation.

Each hidden layer contains multiple neurons. The neuron format is shown in Figure 10. The number of input X and output Y is set as required, but X_0_ is the specified value −1. Each input corresponds to a weight win, and X_0_ corresponds to w_0_θ. In the calculation process, the sum is first and then the mapping is performed.

Where X and W are shown in Equation (9).

(9) $$X=\left[X_{0},X_{1},\ldots ,X_{n}\right],W=\left[\begin{array}{c} w_{i0}\\ w_{i1}\\ \vdots \\ w_{\mathrm{in}} \end{array}\right]$$

(10) $$\mathrm{So}\;{\mathrm{net}}_{i}=\overset{n}{\underset{j=1}{\sum }}W_{\mathrm{ij}}X_{j}-\theta =\mathrm{XW}$$

Thus, the output can be represented as:

(11) $$y_{i}=f({\mathrm{net}}_{i})=f(\mathrm{XW})$$

**Figure 11:**
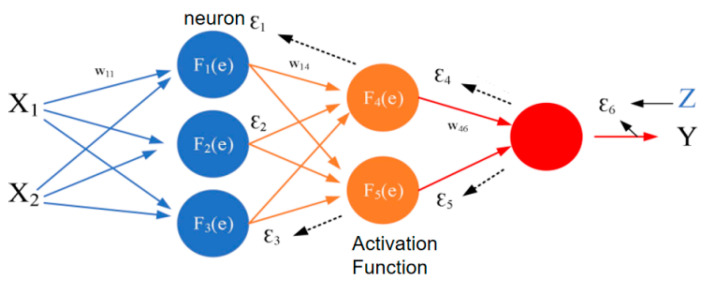
Back propagation neural network construction reference.

In this way, the calculation of one neuron is completed. The reference for the construction of the entire BP neural network model is shown in Figure 11.

The result of each layer of neurons is the sum of the products of the previous layer and the weights. We continued in turn until the predicted value Y was output, and then compared it with the actual value. An error ε6 was generated. F6(e) pushed the error backwards, and Errors ε4 and ε5 were formed in F4(e) and F5(e) in turn. The error backward calculation is shown in Equations (12) and (13).

(12) $$\varepsilon_{4}=W_{46}\varepsilon_{6}$$

(13) $$\varepsilon_{1}=W_{14}\varepsilon_{4}+W_{15}\varepsilon_{5}$$

In this way, the errors of all levels were calculated backward in turn. Then, we started from the first layer to adjust the weights of all levels to reduce errors. Then, we calculated forward, and repeated the operation until the error with the actual value was between the set value. At this point, the constructed model is the established BP neural network model. The number of neurons in the model and the setting of the training function are the keys to affecting the accuracy of the model.

### 3.8. Model Evaluation Metrics

After the prediction model is established, the fitting effect and prediction effect of the model should be evaluated. In order to obtain modeling results, evaluation metrics should be applied when modeling. The models were evaluated and compared through the evaluation indicators to select the optimal model.

In this paper, model evaluation indicators such as root mean square error (RMSE), mean squared error (MSE), and coefficient of determination (R^2^) in statistics were used to evaluate the model. The formula is as follows: (14), (15), (16). The following will introduce these several model evaluation methods, respectively.

- Mean square error:

(14) $$\mathrm{MSE}=\frac{1}{n}\left(\sum_{i=1}^{n}{\left(y_{i}-{\hat{y}}_{i}\right)}^{2}\right)$$

$y_{i}$ refers to the measured value of the component content. ${\overset{\wedge }{y}}_{i}$ refers to the predicted value of the component content. The root mean square error refers to the expected value of the square of the difference between the measured value and the predicted value of the component content.

- Root Mean Square Error:

RMSE refers to the error between the measured value and the predicted value of the component content. The RMSE value becomes smaller, the model effect becomes better, and the prediction accuracy becomes higher. The minimization of the RMSE index value was taken as the optimal parameter to set the optimization goal.

(15) $$\mathrm{RMSE}=\sqrt{\frac{1}{n}\sum_{i=1}^{n}{\left(y_{i}-{\hat{y}}_{i}\right)}^{2}}$$

Among them, n is the number of samples in the data set, $y_{i}$ is the measured value of the component content, and ${\overset{\wedge }{y}}_{i}$ is the predicted value of the component content. Root mean square error of calibration (RMSEC) refers to the root mean square error of the modeling set. Root mean square error of prediction (RMSEP) refers to the root mean square error of the test set.

- Determination coefficient R^2^:

The coefficient of determination R^2^ refers to the fitting effect between the predicted value and the measured value. R^2^_C_ refers to the model set cross-validation coefficient of determination, and R^2^_P_ refers to the test set coefficient of determination.

(16) $$R^{2}=\frac{\sum_{i=1}^{n}{\left(\overset{\wedge }{y}-{\bar{y}}_{i}\right)}^{2}}{\sum_{i=1}^{n}{\left(y-{\bar{y}}_{i}\right)}^{2}}$$

In the formula, n refers to the number of samples, $y_{i}$ is the measured value of the component, ${\overset{\wedge }{y}}_{i}$ is the predicted value of the model, and ${\bar{y}}_{i}$ is the average value of the measured value. The closer the coefficient of determination R^2^ is to 1, the higher the fitting degree of the algorithm model and the better the model modeling effect. The closer R^2^ is to 0, the worse the fitting degree of the model and the poorer the modeling effect.

## 4. Results and Discussion

### 4.1. RF Modeling

The modeling data adopt the 100 samples data randomly sampled earlier. The data statistics of the modeling set are shown in Table 4.

Among the 100 soil samples in the modeling set, the nitrogen content was distributed between 0.655 and 2.104 g/kg; the standard deviation was 0.331 g/kg.

**Figure 12:**
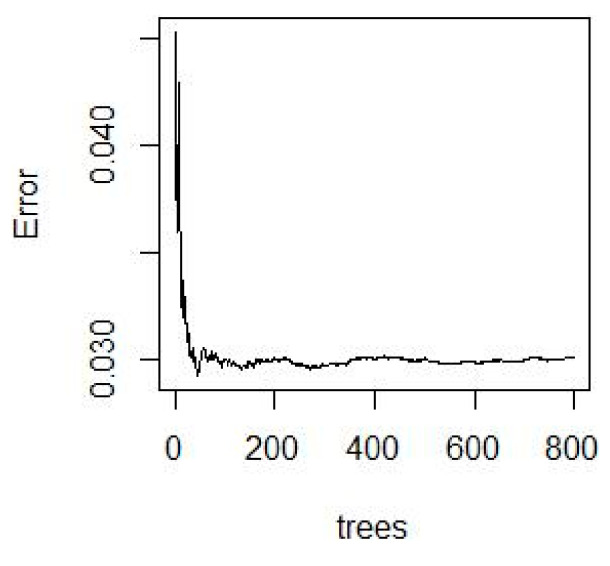
Nitrogen content error rate varies with tree number.

Spectral data and the measured nitrogen content from simulated soil samples were incorporated into random forest models. The MSE error rate spectrum corresponding to the parameter Ntree was obtained using a cross-validation method. The spectral lines are shown in Figure 12.

**Figure 13:**
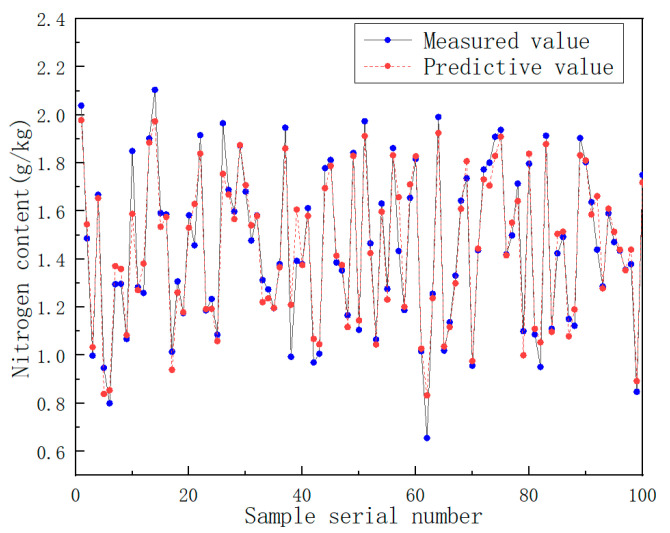
Comparison of predicted and measured nitrogen content in random forest model.

As can be seen from Figure 12, the larger the value of Ntree, the more stable the corresponding random forest model. It also shows that RF is robust to overfitting. Different combinations of the number of regression trees and the number of split nodes were chosen. The value of the number of regression trees was kept unchanged, so that the number of split nodes could be debugged within the optional range. Then, the best parameter value was selected. The number of nitrogen content trees, the number of split nodes, and the RMSEC statistics are shown in Table 5. The comparison of the predicted and measured values of model nitrogen content can be seen in Figure 13.

There are a total of 43 soil samples in the test set. The statistics of the measured values of soil samples in the test set are shown in Table 6. The distribution of nitrogen content is 0.609~2.092 g/kg, and the standard deviation is 0.332 g/kg.

**Figure 14:**
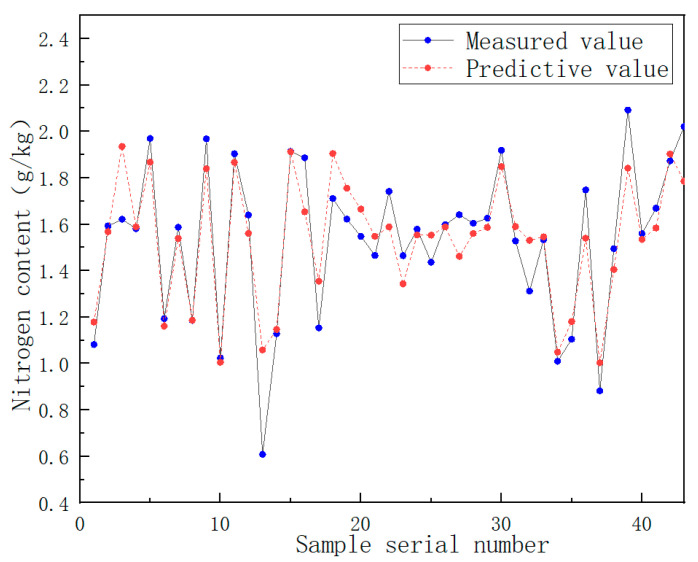
Comparison of the predicted value of nitrogen content in the test set with the measured value.

To verify the feasibility of detecting soil components using the near-infrared spectrum and random forest algorithm, the nitrogen random forest prediction model was adjusted to the optimal model parameters. The spectral data of 43 soil samples in the test set were brought into the random forest model. It was concluded that the coefficient of determination (R^2^_P_) for the prediction of the nitrogen content in the test set was 0.83, and the root mean square error (RMSEP) was 0.141. The comparison between the predicted value of the test set component content and the measured value is shown in Figure 14.

### 4.2. Analysis of Experimental Results

**Figure 15:**
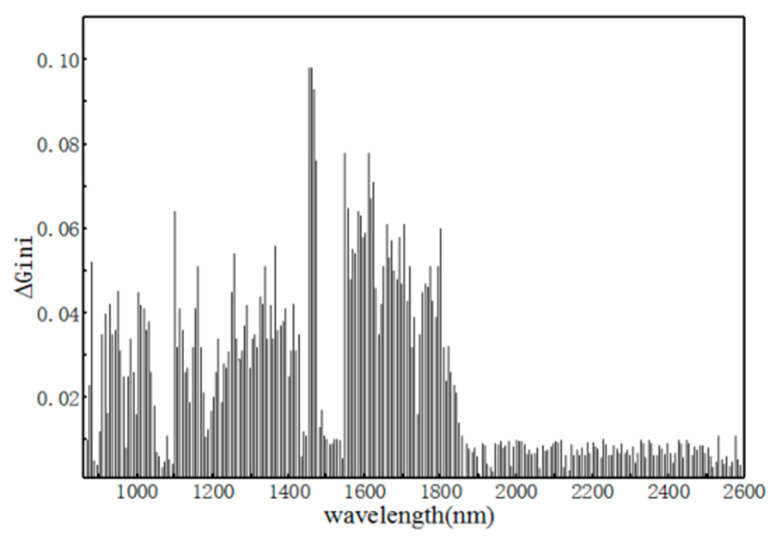
Nitrogen content of soil samples based on random forest with 256-wavelength ∆Gini variation.

The RF regression optimal parameter (Ntree, NSV) for adjusting the soil nitrogen content is (300, 50). The cross-validation result R^2^_C_ is 0.921 and the RMSEC is 0.115. In the process of splitting and growing the decision tree, the Gini coefficients before and after each split node were obtained. Then, the mean of all ΔGini values for each wavelength of the 300 decision trees was calculated as an indicator of the importance of the wavelength to the component content. The nitrogen content of soil samples based on 256 wavelengths ΔGini of RF modeling is shown in Figure 15. The importance of all wavelengths was further determined by the size of ΔGini: the larger the ΔGini, the more information the wavelength contains.

As can be seen from Figure 15, the wavelength is more important between 860 and 1900 nm. The first 148 wavelengths with a higher ΔGini were selected as the preferred wavelengths. A random stand prediction model was established based on the optimal wavelength characteristics The R^2^_C_ accuracy of the built model is 0.918 and the RMSEC accuracy is 0.119. The results are similar to the full-wave modeling result. It was demonstrated that the near-infrared spectrum associated with the soil nitrogen content can be optimized by ΔGini.

Combined with Figure 15 and Table 7, it indicates that the spectral information related to the soil N content can be extracted by optimizing RF modeling data based on ΔGini. This also shows that the optimal features based on ∆Gini can reduce redundant data and thus can simplify the model, thereby optimizing the model prediction speed.

## 5. Comparison of Different Models

Support vector machines [44,45,46] (SVM) and neural networks [47,48,49] (BP) are two common high-precision modeling algorithms in the study of soil composition detection using near-infrared spectroscopy. The superiority of the two algorithms has been highlighted in many near-infrared spectroscopy detection literatures.

To further test the performance of the model, SVM algorithm and BP neural network modeling commonly used in the detection of soil components by near-infrared spectroscopy were selected for comparison.

### 5.1. Support Vector Machine Modeling

The preprocessed spectral data and the measured soil nitrogen content were predicted using SVM modeling with different kernel functions. The parameters were optimized and tuned. Five-time cross validation was applied to obtain the parameters of the model evaluation. The results are shown in Table 8.

**Figure 16:**
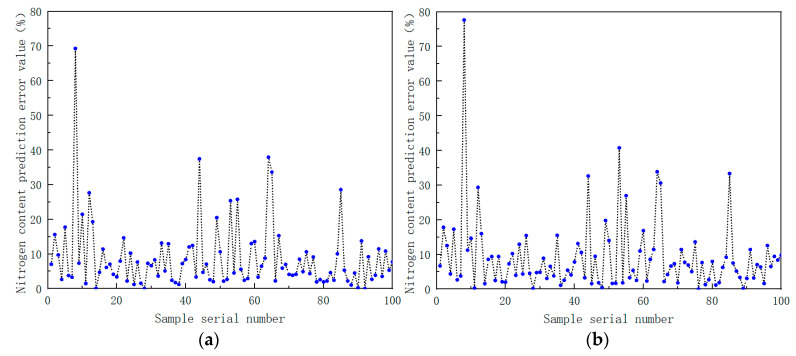
(**a**) Prediction results of soil nitrogen content using support vector machine model based on linear kernel function; (**b**) prediction results of soil nitrogen content using support vector machine model based on Gaussian kernel function.

From Table 8, in terms of the SVM modeling results of the soil nitrogen content, the SVM modeling results based on the linear kernel function are the best. Its model determination coefficient R^2^ is 0.78, and its root mean square error RMSE is 0.156. The best linear and Gaussian SVM modeling prediction results are shown in Figure 16. In the scatter plot, it can also be seen that the prediction results of the linear SVM model are closer to the measured results, and the prediction effect is the best.

### 5.2. BP Neural Network Modeling

**Figure 17:**
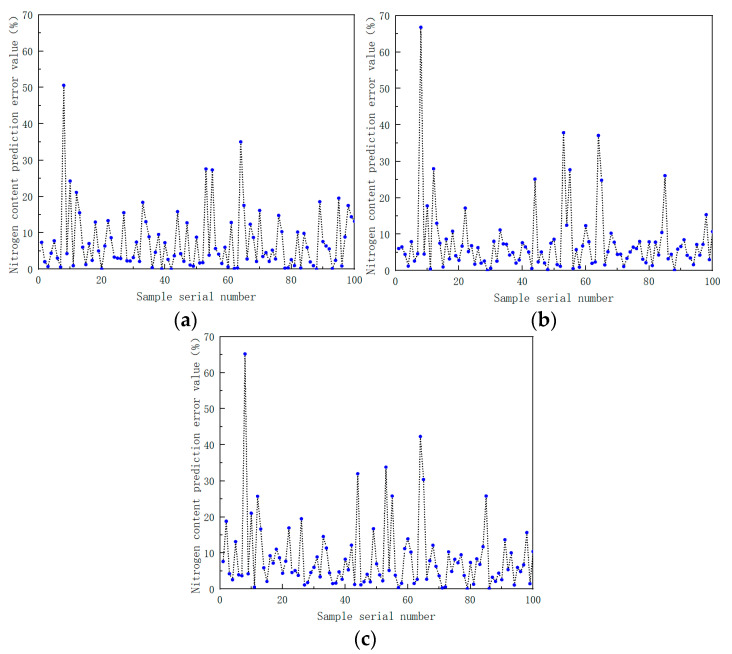
(**a**) Prediction results of soil nitrogen content using back propagation neural network based on Levenberg–Marquardt training function; (**b**) prediction results of soil nitrogen content using back propagation neural network based on Bayesian regularization training function; (**c**) prediction results of soil nitrogen content using back propagation neural network based on scaled conjugate gradient training function.

The preprocessed spectral data were used as the input of the BP neural network, and the measured value of the soil nitrogen content was used as the output of the BP neural network. The number of hidden neurons was adjusted and different training functions were chosen in order to choose the optimal modeling parameters. The modeling results are shown in Table 9. The percentage of error between the model predicted value and the measured value is shown in Figure 17.

From Table 9, it is clear that, when the BP neural network adopts the Levenberg–Marquardt training model, and the number of neurons is 18, the model is the best. The model determination coefficient R^2^ is 0.876 and the root mean square error is 0.111. In the model prediction scatterplot, it can also be seen that the BP neural network based on the Levenberg–Marquardt training function has a closer correlation with the prediction results of the soil nitrogen content and the measured results. The prediction effect is the best.

From the results of the RMSE and R^2^ index data in Table 10 and the error values of the model prediction results in Figure 17, the following conclusions can be drawn. In the modeling of each component, compared with the BP and SVM models, the random forest model RF prediction results are closer to the measured results, and the prediction effect is better. At the same time, it further verified the feasibility and superiority of forest-based near-infrared spectroscopy for the detection of various soil components.

## 6. Conclusions

In this paper, the 143 spectral data obtained from the experiment were smoothed and denoised. The moving average (movmean), Gaussian filter (Gaussian), moving median (movmmedian), local weighted regression (lowess), local polynomial regression fitting (loess), robust local weighted regression (rlowess), robust local polynomial regression (rloess), least squares smoothing filter (sgolay) data spectrogram, and root mean square error results were compared. The local polynomial regression fitting (loess) method has the best smoothing effect. The local polynomial regression fitting (loess) method can also eliminate noise and reduce the error of the component prediction results.

Based on the near-infrared spectrum of soil and the measured value of the nitrogen content, a random forest prediction model was established. The parameters were adjusted to obtain the model with the best prediction effect. Firstly, the pre-processed spectrum data were used as the input of the model, and then a random forest regression model was set up. The number of CART regression trees (Ntree), the number of split nodes (NSV), and the MSE results were adjusted to optimize the parameters of the random forest model. The wavelength was optimized by ΔGini, the data dimension was reduced, and the result was compared with the full wave. The results show that, when the parameters of the nitrogen content prediction model based on a random forest are at (300, 50), the 860–1900 nm band is the most suitable for modeling. At this point, the model cross-validation R^2^_C_ is 0.918, and the RMSEC is 0.119. It is also proved that the optimal wavelength of ΔGini can be optimized for the near-infrared spectrum associated with the soil nitrogen content, which can reduce redundant information of data and optimize the model.

The optimum prediction model of the soil nitrogen content was validated. The spectral data and component measured values of the 43 soil samples used as the verification part were measured, and the local polynomial regression fitting (loess) method was used to smooth and denoise the spectral data. The optimal model was selected for prediction. The results show that: the RF model modeling set prediction R^2^_C_ is 0.921, the RMSEC is 0.115, the test set R^2^_P_ is 0.83, and the RMSEP is 0.141. The prediction of soil components is very close to the measured value. Therefore, the feasibility of predicting the nitrogen content in soil components using the optimal model based on a random forest is verified.

The soil composition detection model based on the near-infrared spectrum was established with common neural network (BP) and support vector machine (SVM) algorithms. The optimal BP and SVM models were selected by adjusting the model parameters. By comparing the two evaluation indicators of the root mean square error (RMSE) and correlation coefficient (R^2^) with the random forest, the RF model has a better accuracy and prediction effect than the BP and SVM models. The feasibility and superiority of soil multi-component detection based on RF and NIR spectroscopy technology were further verified.

## Figures and Tables

**Table 1:** Instrument parameter table.

Instrument	Parameter	
	Integration time	1~400 ms
NIR Quest (256–2.5)	Wavelength range	900~2500 nm
	Optical fiber connector	SMA905
	Wavelength range	360~2400 nm
Tungsten halogen lamps	Output range	8.8 mA
	Color temperature	3000 K

**Table 2:** Experimental parameter setting table.

Name	Parameters
Detection wavelength range	860~2600 nm
Resolution	7 nm
Number of scans	10 times
Laboratory temperature	24~26 °C

**Table 3:** Experimental parameter setting table.

Smoothing Method	RMSE
movmean	3.14
Gaussian	1.90
movmmedian	3.25
Lowess	1.93
loess	0.84
Rlowess	2.66
Rloess	1.68
Sgolay	1.49

**Table 4:** Statistical table of component content of soil pond sample modeling set.

Ingredient Category	Number of Samples	Average Value	Standard Deviation
nitrogen	100	1.445	0.331

**Table 5:** Nitrogen content tree number and split node number setting and RMSEC statistical graph.

Ntree Fetching Values	Optimal Number of Split Nodes	RMSEC
800	80	0.117
600	90	0.116
500	70	0.117
400	60	0.118
300	50	0.115
150	70	0.117

**Table 6:** Soil sample prediction set component content statistics table.

Ingredient Category	Number of Samples	Average Value	Standard Deviation
nitrogen	43	1.531	0.332

**Table 7:** Comparison of preferred wavelength and full-wavelength-based RF modeling results.

Models	Wave Length	RMSEC	R^2^_C_	RMSEP	R^2^_P_
RF	148	0.1178	0.951	0.1453	0.899
RF	256	0.1155	0.957	0.1412	0.909

**Table 8:** Modeling results of soil nitrogen content with different kernel functions SVM.

Model	Kernel Function	RMSE	R^2^
SVM	linear	0.156	0.78
	secondary	0.280	0.30
	Gaussian	0.161	0.76

**Table 9:** BP neural network modeling results based on different training models.

Model	Training Function	Neurons	RMSE	R^2^
BP	Levenberg–Marquardt	18	0.111	0.876
	Bayesian Regularization	18	0.132	0.835
	Scaled Conjugate Gradient	18	0.124	0.851

**Table 10:** Comparison of prediction performance of different models.

Serial Number	Model	RMSE	R^2^
1	SVM (linear)	0.156	0.780
2	BP (Levenberg–Marquardt)	0.111	0.876
3	random forest	0.116	0.921

## Data Availability

Not applicable.

## References

[1] Ma Z., Zhang K., Bai J., Wang J., Chen W. (2021). Effects of different water and nitrogen dosages on tomato growth and soil environment in the root zone under facility conditions. Hubei Agric. Sci..

[2] Liu Y. (2020). Method Improvement of Kjeldahl Method for Determination of Total Nitrogen in Soil Quality. Chem. Manag..

[3] Zhang X., Nv S. (2018). Determination of total nitrogen in soil—Modified Kjeldahl method. Agric. Technol..

[4] Su R., Wu J., Hu J., Ma L., Ahmed S., Zhang Y., Abdulraheem I., Birech Z., Li L., Li C. (2022). Minimalizing Non-Point Source Pollution Using a Cooperative Ion Selection Electrode System for Estimating Nitrate Nitrogen in Soil. Front. Plant Sci..

[5] Chen S., Jiang Q., Wang K. (2011). Application and research progress of remote sensing in estimation of soil organic matter content. J. Shandong Agric. Univ..

[6] Li B., Shen R., Yan J., Liu L., Huang X. (2013). Research on Soil Resistivity Estimation Based on Remote Sensing. J. Nanjing Univ. Inf. Technol..

[7] Mo X., Li H., Lian Y., Lian Y., Zheng B., Dong J., Lu X. (2021). Estimation of soil microplastic input derived from plastic gauze using a simplified model. Sci. Total Environ..

[8] Odebiri O., Mutanga O., Odindi J., Naicker R., Masemola C., Sibanda M. (2021). Deep learning approaches in remote sensing of soil organic carbon: A review of utility, challenges, and prospects. Environ. Monit. Assess..

[9] Wang J., Tao F., Guo S., Ye S. (2021). Application progress of near-infrared spectroscopy in food safety detection. Food Ind..

[10] Pandiselvam R., Mahanti N.K., Manikantan M.R., Kothakota A., Chakraborty S., Ramesh S., Beegum P. (2022). Rapid detection of adulteration in desiccated coconut powder: Vis-NIR spectroscopy and chemometric approach. Food Control.

[11] Jiang H., Lin H., Lin J., Chen Q., Xue Z., Chan C. (2022). Non-destructive detection of multi-component heavy metals in corn oil using nano-modified colorimetric sensor combined with near-infrared spectroscopy. Food Control.

[12] Franzoi M., Costa A., Goi A., Penasa M., Marchi D. (2022). Effectiveness of visible–Near infrared spectroscopy coupled with simulated annealing partial least squares analysis to predict immunoglobulins G, A, and M concentration in bovine colostrum. Food Chem..

[13] Huang Y., Du G., Ma Y., Zhou J. (2021). Predicting heavy metals in dark sun-cured tobacco by near-infrared spectroscopy modeling based on the optimized variable selections. Ind. Crops Prod..

[14] Zhang F., Gen X., Li M., Hu l., Zhou K. (2021). Rapid detection of fiber content in cotton/spandex blended fabrics based on near-infrared spectroscopy. Infrared.

[15] Liu S., Wang S., Hu C., Bi W. (2022). Determination of alcohols-diesel oil by near infrared spectroscopy based on gramian angular field image coding and deep learning. Fuel.

[16] Chen S., Nao W., Xv Z., Liu Q., Wen H., Deng H. (2014). Fourier Transform Infrared Spectroscopy Detection of Breast Cancer Related Research. J. Zhejiang Univ..

[17] Han G., Wang X., Chen S., Wang H., Wang J., Zhao Z. (2021). Research progress on improving the accuracy of near-infrared spectroscopy in the detection of complex solutions such as human blood. Spectrosc. Spectr. Anal..

[18] Gao M., Zhang Y., Cheng F., Wang H., Liu L., Jin X., Zhou Y., Wang T., Chen P., Yao W. (2022). A gradient-based discriminant analysis method for process quality control of carbonized TCM via Fourier transform near infrared spectroscopy: A case study on carbonized Typhae Pollen. Spectrochim. Acta Part A Mol. Biomol. Spectrosc..

[19] Losso K., Bec K.B., Mayr S., Grabska J., Stuppner S., Jones M., Jakschitz T., Rainer M., Bonn K., Huck W. (2022). Rapid discrimination of Curcuma longa and Curcuma xanthorrhiza using Direct Analysis in Real Time Mass Spectrometry and Near Infrared Spectroscopy. Spectrochim. Acta Part A Mol. Biomol. Spectrosc..

[20] Tan B., Xiao T., Li G., Huang C. (2020). Study of Nondestructive Detection of Fruit Near-infrared Diffuse Reflection Experiment and its Spectrum Data Analysis. Basic & Clinical Pharmacology & Toxicology.

[21] Chen H., Liu Z., Gu J., Ai W., Wen J., Cai K. (2018). Quantitative analysis of soil nutrition based on FT-NIR spectroscopy integrated with BP neural deep learning. Anal. Methods.

[22] Qiao H., Shi X., Chen H., Lu S., Hong S. (2022). Effective prediction of soil organic matter by deep SVD concatenation using FT-NIR spectroscopy. Soil Tillage Res..

[23] Tan B., Xiao T., Liu Q., Li G., Li G., Huang C. (2021). Experiment and analysis of near-infrared diffuse reflection detection of sugar content in fruit. J. Cent. China Norm. Univ..

[24] Zheng B., Xiao T., Wang M., Tian S., Tan B. (2022). Soil Nitrogen Detection Based on Random Forest Algorithm and Near Infrared Spectroscopy. Proceedings of the 2022 International Conference on Computation, Big-Data and Engineering (ICCBE).

[25] O’Hara R., Zimmermann J., Green S. (2021). A multimodality test outperforms three machine learning classifiers for identifying and mapping paddocks using time series satellite imagery. Geocarto Int..

[26] Bedon L., Cecchin E., Fabbiani E., Michele Dal B., Buonadonna A., Polano M. (2021). Machine Learning Application in a Phase I Clinical Trial Allows to Identify Clinical-Biomolecular Markers Significantly Associated with Toxicity. Clin. Pharmacol. Ther..

[27] Li S., Jia M., Dong D. (2018). Near-infrared spectroscopy measurement of fruit sugar by random forest algorithm. Spectrosc. Spectr. Anal..

[28] Li M., Xue J., Du Y., Zhang T., Li H. (2019). Data Fusion of Raman and Near-Infrared Spectroscopies for the Rapid Quantitative Analysis of Methanol Content in Methanol–Gasoline. Energy Fuels.

[29] Kartakoullis A., Comaposada J., Cruz-Carrión A., Serra X., Gou P. (2019). Feasibility study of smartphone-based Near Infrared Spectroscopy (NIRS) for salted minced meat composition diagnostics at different temperatures. Food Chem..

[30] Liu Z., Wen J., Gao H., Ding H. (2017). Research on near infrared modeling of soil organic matter based on random forest method. Mod. Agric. Equip..

[31] Fang X., Zhang H., Hang L., He Y. (2015). Study on the detection of soil total nitrogen by near infrared spectroscopy. Spectrosc. Spectr. Anal..

[32] Xue Y., Ping P., Guang L., Lu M. (2017). Rapid detection of soil nutrients based on visible and near infrared spectroscopy. Spectrosc. Spectr. Anal..

[33] Fang X., Wang W., Jin X., Li C. (2019). Visible-near infrared spectroscopy detection method of soil available phosphorus. Jiangsu J. Agric. Sci..

[34] Wang Y., Huang H., Chen X. (2021). Predicting organic matter content, Total nitrogen and pH value of lime concretion black soil based on visible and near infrared spectroscopy. Eurasian Soil Sci..

[35] Xu F., Fu D., Wang Q., Xiao Z., Wang B. (2018). A visible NIR spectroscopy method for Non-Destructive detection of sugar and acidity in red extract based on MCCV-CARS-RF. Food Sci..

[36] Liu J., Sun S., Tan Z., Liu Y. (2020). Nondestructive detection of sunset yellow in cream based on near-infrared spectroscopy and interval random forest. Spectrochim. Acta Part A Mol. Biomol. Spectrosc..

[37] Du C., Sun L., Bai H., Liu Y., Yang J., Wang X. (2021). Quantitative detection of azodicarbonamide in wheat flour by near-infrared spectroscopy based on two-step feature selection. Chemom. Intell. Lab. Syst..

[38] Shahrayini E., Noroozi A.A., Eghbal M.K. (2020). Prediction of Soil Properties by Visible and Near-Infrared Reflectance Spectroscopy. Eurasian Soil Sci..

[39] Cui Y., Yang W., Wang W., Wang D., Meng C., Li M. (2021). Design and experiment of portable soil organic matter detector based on spectroscopy principle. Trans. Chin. Soc. Agric. Mach..

[40] Luo D., Peng J., Feng C., Liu W., Ji W., Wang N. (2021). Retrieval of soil organic matter components in Visible-Near-Infrared and Mid-Infrared spectra. Spectrosc. Spectr. Anal..

[41] Hong Y., Chen Y., Shen R., Chen S., Gang X., Hang C., Guo Z., Wei Z., Yang J., Liu Y. (2021). Diagnosis of cadmium contamination in urban and suburban soils using visible-to-near-infrared spectroscopy. Environ. Pollut..

[42] Wang H., Zeng H. (2006). Performance Analysis of Several Image Denoising and Smoothing Algorithms. Sci. Technol. Inf..

[43] Yang M. (2020). A Near-Infrared Spectroscopic Method for the Detection of Components in the Fermentation Process of Ethanol and Monosodium Glutamate. Master’s Thesis.

[44] de Santana F.B., Otani S.K., de Souza A.M., Poppi R. (2021). Comparison of PLS and SVM models for soil organic matter and particle size using vis-NIR spectral libraries. Geoderma Reg..

[45] Gholizadeh A., Coblinski J.A., Saberioon M., Ben-Dor E., Drábek O. (2021). Vis–NIR and XRF data fusion and feature selection to estimate potentially toxic elements in soil. Sensors.

[46] Gholizadeh A., Rossel R.A.V., Saberioon M., Boruvka L., Kratina J. (2021). National-scale spectroscopic assessment of soil organic carbon in forests of the Czech Republic. Geoderma.

[47] Wu Q., Yang Y., Xu Z., Jin Y., Guo Y., Lao C. (2014). Applying local neural network and visible/near-infrared spectroscopy to estimating available nitrogen, phosphorus and potassium in soil. Spectroscopy and spectral analysis.

[48] Tang Y., Chen Z. (2021). Soil pH Prediction Based on Convolution Neural Network and Near Infrared Spectroscopy. Spectrosc. Spectr. Anal..

[49] Wojcik G., Hubicki Z., Rusek P. (2020). A Back Propagation Neural Network Model Optimized by Mind Evolutionary Algorithm for Estimating Cd, Cr, and Pb Concentrations in Soils Using Vis-NIR Diffuse Reflectance Spectroscopy. Appl. Sci..

